# Cardiovascular magnetic resonance techniques and findings in children with myocarditis: a multicenter retrospective study

**DOI:** 10.1186/s12968-015-0201-6

**Published:** 2015-11-17

**Authors:** Puja Banka, Joshua D. Robinson, Santosh C. Uppu, Matthew A. Harris, Keren Hasbani, Wyman W. Lai, Marc E. Richmond, Sohrab Fratz, Supriya Jain, Tiffanie R. Johnson, Shiraz A. Maskatia, Jimmy C. Lu, Margaret M. Samyn, David Patton, Andrew J. Powell

**Affiliations:** Boston Children’s Hospital and Harvard Medical School, 300 Longwood Ave, Boston, MA 02115 USA; Ann & Robert H Lurie Children’s Hospital of Chicago and Northwestern University Feinberg School of Medicine, Chicago, IL USA; Mount Sinai Hospital and Icahn School of Medicine, New York, NY USA; Children’s Hospital of Philadelphia and Perelman School of Medicine, Philadelphia, PA USA; Advocate Children’s Hospital, Park Ridge, IL USA; Morgan Stanley Children’s Hospital of NewYork-Presbyterian and Columbia College of Physicians and Surgeons, New York, NY USA; Deutsches Herzzentrum München, Munich, Germany; Maria Fareri Children’s Hospital at Westchester Medical Center and New York Medical College, Valhalla, NY USA; Riley Hospital for Children and Indiana University school of Medicine, Indianapolis, IN USA; Texas Children’s Hospital and Baylor College of Medicine, Houston, TX USA; C. S. Mott Children’s Hospital and University of Michigan Medical School, Ann Arbor, MI USA; Children’s Hospital of Wisconsin and Medical College of Wisconsin, Milwaukee, WI USA; Alberta Children’s Hospital and University of Calgary, Calgary, AB Canada

**Keywords:** Cardiovascular magnetic resonance, Myocarditis, Pediatrics, Late gadolinium enhancement, Early gadolinium enhancement, T2-weighted imaging

## Abstract

**Background:**

Cardiovascular magnetic resonance (CMR) is increasingly used to diagnose myocarditis in adults but its use in children is not well-established. We sought to describe the presentation, CMR protocol and findings, and outcomes in a multicenter cohort of children with myocarditis.

**Methods:**

Thirteen hospitals retrospectively identified patients meeting the following inclusion criteria: 1) diagnosis of myocarditis by the managing physicians, 2) age <21 years, 3) CMR examination within 30 days of presentation, and 4) no congenital heart disease. Clinical data and test results, including CMR findings, were abstracted from the medical record.

**Results:**

For the 143 patients meeting inclusion criteria, the median age was 16.0 years (range, 0.1-20.3) and 139 (97 %) were hospitalized at the time of CMR. The median time from presentation to CMR was 2 days (0-28). The median left ventricular ejection fraction at CMR was 56 % (10-74), with 29 (20 %) below 45 %. The median right ventricular ejection fraction was 54 % (15-72), with 11 (8 %) below 40 %. There was significant variability among centers in the types of tissue characterization techniques employed (*p <* 0.001). Overall, late gadolinium enhancement (LGE) was used in 100 % of studies, followed by T2-weighted imaging (T2W) in 69 %, first-pass contrast perfusion (FPP) in 48 %, and early gadolinium enhancement (EGE) in 28 %. Abnormalities were most common with LGE (81 %), followed by T2W (74 %), EGE (55 %), and FPP (8 %). The CMR study was interpreted as positive for myocarditis in 117 patients (82 %), negative in 18 (13 %), and equivocal in 7 (5 %), yielding a sensitivity of 82 %. At a median follow-up of 7.1 months (0-87), all patients were alive and 5 had undergone cardiac transplantation. CMR parameters at presentation associated with persistent left ventricular dysfunction were larger left ventricular end-diastolic volume and lower left and right ventricular ejection fraction but not abnormal LGE.

**Conclusions:**

Despite significant practice variation in imaging protocol among centers, CMR had a high sensitivity for the diagnosis of myocarditis in pediatric patients. Abnormalities were most often seen with LGE followed by T2W, EGE, and FPP. These findings should be useful in designing future prospective studies.

## Backgound

Viral myocarditis is an important cause of morbidity and mortality in both children and adults [1–4]. It may lead to acute heart failure, dilated cardiomyopathy, and sudden cardiac death. The accurate diagnosis of myocarditis is challenging because the severity and type of symptoms is quite variable. Moreover, no single test can confirm or exclude the diagnosis with certainty. Endomyocardial biopsy, the most widely accepted standard, still suffers from sampling errors which reduce sensitivity [5], suboptimal interobserver agreement [6], and the risk of complications [7, 8]. Thus, in practice, history, test results, and clinical course are all integrated to make the diagnosis.

Over the past decade, cardiovascular magnetic resonance (CMR) has emerged as an important noninvasive tool for the diagnosis and monitoring of myocarditis in adults [9, 10]. In addition to providing reliable measurements of ventricular size and function, CMR myocardial tissue characterization techniques can assess for inflammatory changes such as edema, hyperemia, capillary leak, and myocyte necrosis [11]. As a result, the use of CMR in evaluating patients with known or suspected myocarditis was deemed “appropriate” by a multi-society consensus group in 2006 [12], and suspected myocarditis has become one of the most common indications for CMR in adults [13, 14]. Even with CMR, there is no single pathognomonic finding. Rather, it is common practice to apply the Lake Louise Criteria which requires abnormalities in 2 of 3 tissue characterization techniques: T2-weighted imaging (T2W) which assesses for edema, T1-weighted early gadolinium enhancement imaging (EGE) which assesses for hyperemia, and late gadolinium enhancement imaging (LGE) which assesses for myocyte necrosis and fibrosis [9].

Although one of the earliest reports on the diagnosis of myocarditis with CMR was in children [15], there are only a few contemporary pediatric studies, and these are all single center reports with relatively small sample sizes [4, 16–18]. We, therefore, sought to describe the clinical presentation, CMR protocols and findings, and outcomes in a large, multicenter cohort of children with myocarditis.

## Methods

### Patients

This was a retrospective, multicenter study. Investigators at the coordinating center (Boston Children’s Hospital) solicited participation from an international group of pediatric centers through the Society for Cardiovascular Magnetic Resonance Pediatric/Congenital Research Working Group. Each center identified all patients who met the following inclusion criteria: 1) ultimate diagnosis of myocarditis by the patient’s managing physicians, 2) age at presentation <21 years, 3) CMR within 30 days of presentation, and 4) no history of congenital heart disease. The study was approved by each center’s institutional review board, all of which waived the requirement for informed consent.

### Data collection

For each patient, centers completed an electronic case information form produced and managed using Research Electronic Data Capture (REDCap) [19] electronic data capture tools that were hosted at Boston Children’s Hospital. REDCap is a Health Insurance Portability and Accountability Act compliant, secure web-based application designed for data collection and management to support clinical and translational research. The following demographic and clinical information were recorded on the form: 1) center name and location; 2) time from presentation to CMR; 3) patient demographic data at the time of CMR (age, weight, height); 4) presenting signs and symptoms; 5) echocardiogram results at presentation, at the time of CMR, and at the latest follow-up; 6) laboratory data; 7) CMR procedural data including scanner type, radiofrequency coil, use of sedation and inotropic support, and sequences; 8) CMR findings at presentation and at follow-up; 9) endomyocardial biopsy results; 10) clinical treatment; 11) time from initial CMR to latest follow-up; and 12) clinical status at latest follow-up.

### CMR image analysis

CMR image analysis and interpretation were done by the performing center in order to best reflect real-world practice. Visual (non-quantitative) assessment was used for late gadolinium enhancement (LGE), first-pass perfusion (FPP), and T2-weighted (T2W) imaging. T1-weighted early gadolinium enhancement (EGE) images were analyzed using myocardial and skeletal muscle signal intensity ratios at 1 of the 8 centers performing this technique; the remainder used visual (non-quantitative) assessment. The classification of each CMR as positive, negative, or equivocal for myocarditis was based on the original interpreting physician’s report. Ventricular dysfunction at CMR was defined as an ejection fraction ≤45 % for the left ventricle (LV) and ≤40 % for the right ventricle (RV).

### Statistical analysis

Continuous variables were summarized as median and range, and categorical variables were described with counts and percentages. Comparisons of the different centers’ use of endomyocardial biopsy and various CMR sequences were conducted using the Pearson Chi-Square test. Ventricular volume and function data between presentation and follow-up CMR studies, as well as between those with and without LV dysfunction at follow-up, and those with and without LGE at follow-up were compared using the Mann-Whitney *U* test. All tests were performed with a 2-sided type I error rate of 0.05. Data analyses were performed using SPSS version 21 (IBM Corporation, USA).

## Results

A total of 143 patients from 13 centers in 3 countries met the study’s inclusion criteria. The number of patients per center ranged from 2 to 33. The patients’ median age was 16.0 years (range 0.1–20.3, interquartile range 13.7,16.9) and all had a diagnosis of myocarditis, as specified by the inclusion criteria. Their clinical data at presentation are summarized in Table 1, and their initial echocardiographic data in Table 2. Of note, 139 patients (97 %) were admitted to the hospital with their initial presentation and had a median stay of 4 days (0-210). Among these, 77 patients (54 %) were admitted to the intensive care unit with a median stay there of 2 days (0-30).

**Table 1:** Subject demographic and clinical data at presentation (n = 143)

	Number (%) or median (range)
Median age (years)	16.0 (0.1 – 20.3)
Symptoms	142 (99 %)
Chest pain	106 (74 %)
Recent or current viral symptoms	60 (42 %)
Shortness of breath	46 (32 %)
Fatigue	35 (25 %)
Fever	34 (24 %)
Palpitations	18 (13 %)
Syncope	11 (8 %)
Poor feeding	10 (7 %)
Signs of congestive heart failure	18 (13 %)
New gallop rhythm	11 (8 %)
New murmur	4 (3 %)
Abnormal electrocardiogram	125 (87 %)
Ventricular tachycardia/fibrillation	12 (8 %)
Atrial tachycardia	2 (1 %)
Abnormal laboratory data	123 (86 %)
Elevated troponin	93 (65 %)
Elevated C-reactive protein	59 (65 %)
Elevated CK-MB	51 (36 %)
Elevated erythrocyte sedimentation rate	18 (13 %)
Admitted to the hospital	139 (97 %)
Inotropic support	36 (25 %)
Ventilatory support	17 (12 %)
Circulatory support	5 (4 %)
Treatment	
Intravenous immunoglobulin	81 (57 %)
Steroids	21(15 %)
Other immunomodulators	8 (6 %)

**Table 2:** Echocardiographic findings at presentation

	Number (%)
Left ventricular function	
Normal	79 (55 %)
Mildly depressed	35 (25 %)
Moderately depressed	15 (11 %)
Severely depressed	14 (10 %)
Left ventricular regional wall motion abnormalities	33 (23 %)
Right ventricular function	
Normal	102 (71 %)
Mildly depressed	5 (4 %)
Moderately depressed	5 (4 %)
Severely depressed	3 (2 %)
Valvar dysfunction (≥moderate)	
Tricuspid regurgitation	4 (3 %)
Mitral regurgitation	13 (20 %)
Pulmonary regurgitation	0 (0 %)
Aortic regurgitation	1 (1 %)
Pericardial effusion	
None/trivial	135 (94 %)
Small	6 (4 %)
Moderate	1 (1 %)

In the cohort, 31 patients (22 %) underwent endomyocardial biopsy, of which 17 were reported as positive for myocarditis, 7 were borderline, and 7 were negative. Viral polymerase chain reaction testing was performed on 14 of the biopsies and was positive in 2. There was no significant difference in the percentage of patients undergoing biopsy among centers (*p* = 0.41).

### CMR

**Fig. 1:**
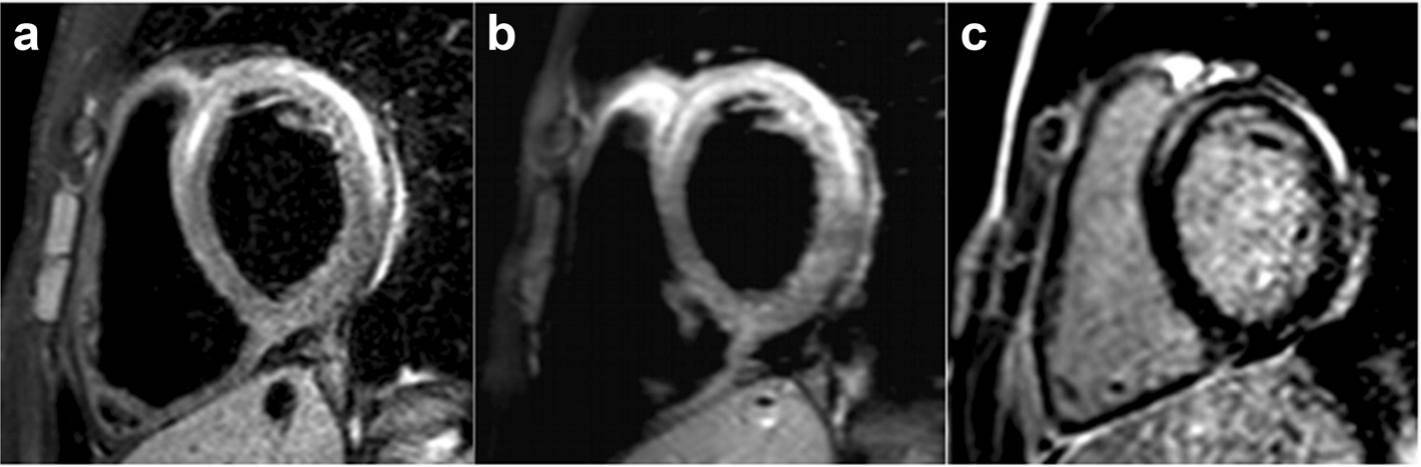
Typical findings of myocarditis on CMR. 16-year-old patient with a midwall and subepicardial distribution of increased signal intensity in the left ventricle on T2-weighted (**a**), T1-weighted early gadolinium enhancement (**b**), and late gadolinium enhancement (**c**) imaging

**Fig. 2:**
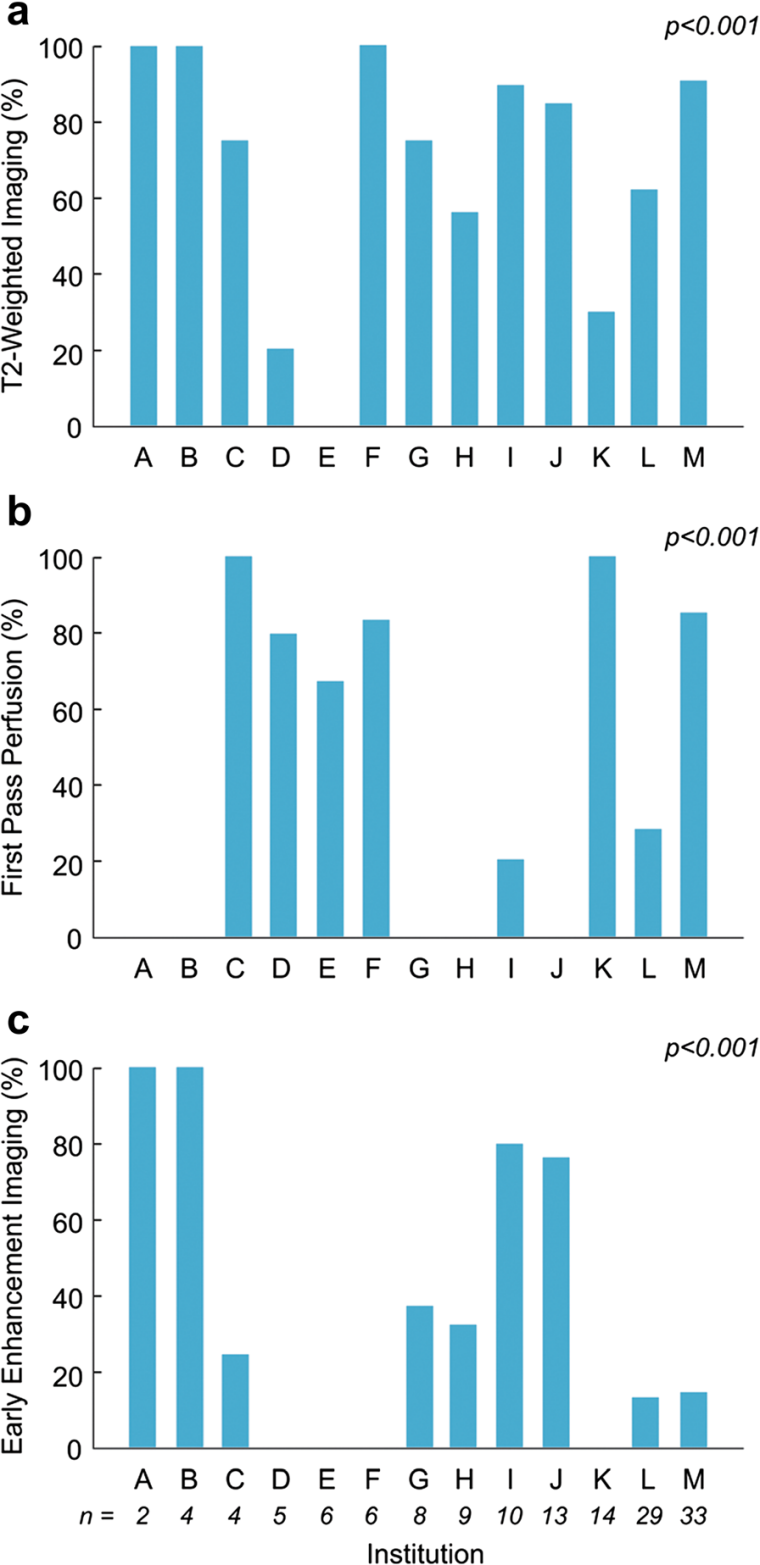
Practice variability in CMR protocols. Histogram showing the percentage of examinations which included T2-weighted (**a**), T1-weighted early gadolinium enhancement (**b**), and late gadolinium enhancement (**c**) imaging at each of the 13 centers. The number of patients enrolled from each center is provided at the bottom of panel **c**

CMR was performed between June 2006 and January 2015, all on a 1.5 Tesla scanner. The median time from presentation to CMR examination was 2 days (range 0-28), with most examinations (137, 96 %) performed within 2 weeks of presentation. Twenty-five examinations (18 %) were done with sedation (median age 7.2 years (0.1-16.8)), and 24 (17 %) occurred with the patient receiving intravenous inotropic medications. The CMR findings for the cohort are summarized in Table 3 and typical images are shown in Fig. 1. All studies included LGE as part of the examination protocol. However, as shown in Fig. 2 and Table 3, there was significant practice variation (*p <* 0.001) among centers in the use of T2W, FPP, and EGE.

**Table 3:** Cardiovascular magnetic resonance findings

	Number (%) or median (range)
Left ventricular end-diastolic volume (ml/m^2^)	87 (38-222)
Left ventricular ejection fraction (%)	56 (10-74)
Left ventricular ejection fraction <45 %	29 (20 %)
Right ventricular end-diastolic volume (ml/m^2^)	87 (44-138)
Right ventricular ejection fraction (%)	54 (15-72)
Right ventricular ejection fraction <40 %	11 (8 %)
Late gadolinium enhancement imaging performed	143 (100 %)
Abnormal	115 (80 %)
Distribution^a^	
Subepicardial	69 (48 %)
Midwall	63 (44 %)
Patchy	9 (6 %)
Subendocardial	6 (4 %)
T2-weighted imaging performed	99 (69 %)
Abnormal	70 (74 %)
First-pass perfusion imaging performed	69 (48 %)
Abnormal	5 (8 %)
Early gadolinium enhancement imaging performed	40 (28 %)
Abnormal	22 (60 %)
Final interpretation regarding myocarditis	
Positive	117 (82 %)
Negative	18 (13 %)
Equivocal	7 (5 %)

^a^Some patients had more than 1 pattern of distribution

LGE was abnormal (i.e., enhancement present) in 115 of 141 patients (81 %) and the most common patterns were subepicardial (48 %) and/or midwall (44 %). T2W was abnormal in 70 of 95 examinations (74 %), EGE was abnormal in 22 of 37 examinations (60 %), and FPP was abnormal in 5 of 65 examinations (8 %). Considering only the 3 techniques which comprise the Lake Louise criteria for a CMR diagnosis of myocarditis (LGE, T2W, and EGE) [9], all 3 were performed in 39 studies, and, among these, 0 techniques were abnormal in 4 patients (11 %), 1 in 6 (15 %), 2 in 13 (33 %), and 3 in 16 (41 %). Thus, 29 of 39 patients (74 %) met the Lake Louise diagnostic standard of having at least 2 of the 3 techniques positive for myocarditis. For all patients, the CMR was interpreted as positive for myocarditis by the reporting physician in 117 patients (82 %), equivocal in 7 (5 %), and negative in 18 (13 %), yielding a sensitivity for an ultimate clinical diagnosis of myocarditis of 82 %.

Among the 17 patients with endomyocardial biopsies positive for myocarditis, LGE was abnormal in 11 of 17, T2W in 3 of 8, and EGE in 2 of 17. Among the 7 patients with negative biopsy results, LGE was abnormal in 6 of 7, T2W in 3 of 4, and EGE in 2 of 2.

### Outcomes

After a median follow-up time from initial CMR of 7.1 months (0-87), all patients were alive and 5 had undergone cardiac transplantation. Among the transplant-free survivors, follow-up echocardiographic data on LV systolic function was available in 112 (78 %). In this group, 44 of the 53 patients (81 %) with LV dysfunction (mild, moderate, or severe) on their presentation echocardiogram had normal LV function at their latest follow-up echocardiogram. Among the 59 patients who had normal function at presentation, 3 (5 %) developed dysfunction at latest follow-up. In other words, 12 patients (11 %) had LV dysfunction at follow-up, 9 of whom had LV dysfunction at presentation and 3 of whom had previously normal function. The findings on the initial CMR that were significantly associated with LV dysfunction at follow-up included larger LV end-diastolic volume (median 110 ml/m^2^ vs. 86 ml/m^2^, *p* = 0.02), lower LV ejection fraction (median 40 % vs. 58 %, *p* = 0.003), and lower RV ejection fraction (median 39 % vs. 55 %, *p* = 0.006). Other CMR parameters, including the age at initial CMR and abnormalities on LGE, T2W, EGE, and FPP, were not significantly associated with LV dysfunction at follow-up.

A follow-up CMR was conducted in 52 patients at a median of 6.0 months (0.2-69) after their initial CMR. The median LV ejection fraction was higher at follow-up CMR (60 % vs. 56 %, *p* = 0.016). LGE was performed in all 52 patients at their follow-up study and was persistently abnormal in 39, persistently negative in 3, changed from abnormal to negative in 6, and changed from negative to abnormal in 4. Among patients in whom T2W was performed at both presentation and follow-up, T2W was persistently abnormal in 6, persistently negative in 5, and changed from abnormal to negative in 24. EGE was persistently abnormal in 2, persistently negative in 3, and changed from abnormal to negative in 4. Patients with abnormal LGE at follow-up had a lower LV ejection fraction (59 % vs. 65 %, *p* = 0.002).

## Discussion

This is the largest published study to date describing the CMR findings in children with myocarditis and the only one that compares CMR protocols across centers. Among the 13 centers in the study, there was significant variability in the types of tissue characterization techniques employed, with LGE being the most common and used by all, followed by T2W, FPP, and EGE. Most examinations were done within 2 weeks of presentation and without sedation. LV ejection fraction at CMR was depressed in 20 % of the patients and RV ejection fraction was depressed in only 8 %. Among the tissue characterization techniques, the highest rate of abnormalities was seen with LGE followed by T2W, EGE, and FPP. Most patients with depressed LV function at presentation recovered normal function. The only CMR parameters at presentation associated with persistent dysfunction were larger LV end-diastolic volume and lower LV and RV ejection fraction.

CMR is now an established technique for the diagnosis of myocarditis in adults [9, 12, 20–22]. Its value stems from tissue characterization capabilities that can detect processes associated with myocardial inflammation. To this end, 3 CMR techniques are now most commonly recommended [9]: 1) T2W for the assessment of intracellular and interstitial edema, 2) EGE for the detection of capillary leakage and hyperemia, and 3) LGE for the visualization of cellular necrosis and subsequent fibrosis. The accuracy of CMR for the diagnosis of myocarditis varies depending on the population being studied, the reference criteria used for a diagnosis of myocarditis (e.g., biopsy), the CMR techniques utilized, and whether a single or a combination of CMR findings is required. Sensitivities and specificities of up to 76 % and 96 %, respectively, have been reported when at least 2 of the above 3 techniques are abnormal and clinical criteria are used as the gold standard [23]. Newer T1 and T2 mapping techniques remove the uncertainty associated with interpreting relative signal intensities and may lead to further improvement in diagnostic performance [16, 24, 25]. CMR can also be helpful for risk stratification to predict those who will have a more benign disease course [26], and to identify patients who are more likely to have adverse events and arrhythmias [21, 27, 28]. Such diagnostic and prognostic data have led to widespread adoption so that suspected myocarditis now accounts for a large proportion of adult cases referred for CMR [14].

In the pediatric population, however, studies on the utility of CMR in acute myocarditis are few. Interestingly, one of the earliest reports on the use of CMR in myocarditis was in children [15]. The authors described increased myocardial to skeletal muscle signal intensity ratios in 6 children with myocarditis compared to 5 without. Since then, despite data suggesting increased use of CMR in children admitted with myocarditis [3], only a few studies have focused on the use of CMR in this population [4, 16–18]. The earliest of these by Kern et al. [18] described LGE in a non-ischemic distribution in 5 children who presented with chest pain and an elevated troponin I. A larger study by Sachdeva et al. [4] reported that half of the 34 children with myocarditis who underwent CMR had LGE. On univariate analysis, LGE was not associated with early or late poor outcomes (mechanical support, heart transplantation, or death); however, on multivariate analysis adjusting for serum brain naturetic peptide, severely decreased ejection fraction, performance of CMR, and serum troponin, LGE was a significant risk factor. In contrast to this study which only described LGE findings, the 2 other reports of CMR in children utilized all 3 of the imaging techniques recommended in the Lake Louise criteria. In 20 patients, Mavrogeni et al. [17] found that 80 % had abnormalities in at least 2 of the 3 techniques, similar to the 74 % in our cohort. Moreover, their patients, like ours, had good outcomes with resolution of LV dysfunction in most patients and no mortality. The other study included 25 children [16] and found that only 36 % fulfilled 2 of 3 criteria. This lower rate of abnormalities might be explained by less severe disease as only 2 of their patients had a LV ejection fraction <55 % and many presented with nonspecific symptoms such as chest pain without elevated serum cardiac enzymes.

In addition to describing the CMR findings in a larger, multicenter population compared to the single center studies mentioned above, our study has important implications with regard to utilization and future research. The relatively high prevalence of CMR abnormalities in children with a clinical diagnosis of myocarditis indicates that it is a sensitive test and supports CMR use when the diagnosis is unclear. This would include patients with new onset chest pain or ventricular arrhythmia in which the clinical history and test results are equivocal for myocarditis. Because our study only included patients with myocarditis, it offers no information regarding the specificity of CMR in children. A prospective investigation in children with suspected myocarditis is thus desirable. Our results provide some insights that should help guide the design of such a study and inform a feasibility assessment. First, a multicenter effort is likely required as no single center performed more than 33 CMR scans in young patients with myocarditis over the entire study period. Second, we found considerable variation in the CMR protocols among the centers. Possible reasons for this variation include unfamiliarity with tissue characterization techniques, which are likely less commonly used with pediatric CMR indications, and the lack of a published protocol specifically for children with suspected myocarditis. Thus, organizers of a prospective study should not assume that centers will be competent performing and interpreting all tissue characterization techniques, and should consider having site education and validation of test studies before enrollment starts. Third, our study found relatively high rates of abnormalities using the 3 techniques most commonly advocated for use in adults with myocarditis—T2W, EGE, and LGE—and thus these should be included in any prospective study. FPP, however, was low-yield and need not be included. Fourth, as endomyocardial biopsy was performed in only 22 % of our cohort, a study design that requires it may be at risk for low enrollment. Finally, in our study cohort, there were no deaths and 5 cardiac transplants, and nearly all of the patients had normal LV function at follow-up. Given this low incidence of poor outcomes, a study aimed at risk stratification will likely need a large number of subjects to be sufficiently powered.

### Limitations

As noted above, this study only included patients who had a clinical diagnosis of myocarditis, precluding an analysis of CMR specificity. This approach was taken because, after careful consideration, the participating centers did not believe they could reliably retrospectively identify all patients with suspected myocarditis who underwent CMR. The final diagnosis of myocarditis in this report was based on the opinion of the patient’s managing physicians. This definition is often used in studies investigating myocarditis [4, 24, 26] because there is no single test with sufficient accuracy, it reflects clinical practice, and it allows multiple factors to be taken into account. However, it is possible that the criteria that were applied might vary by physician and center. Image analysis was based on the interpretation of the original readers, which may vary somewhat among readers and centers. Nevertheless, this approach best reflects real-world practice which was an important aim of this study. Similarly, most centers used visual (non-quantitative) assessment for LGE, FPP, EGE, and T2W imaging which may lead to suboptimal accuracy, particularly for the last 2 of these techniques. Given the small number of patients with LV dysfunction at follow-up, this study may have lacked sufficient power to detect some associations between CMR findings at presentation and persistent LV dysfunction. Finally, the study population likely does not represent the entire clinical spectrum of myocarditis because of referral bias related to CMR. The most severely ill may have been deemed too unstable or unsuitable (e.g., on mechanical circulatory support) to undergo CMR, and those with mild disease may not have engendered sufficient concern to warrant CMR.

## Conclusion

In this largest and only multicenter report on CMR use in children with myocarditis, abnormalities consistent with myocarditis were common. Information from the study on CMR utilization, clinical management, imaging protocols, and outcomes should be useful for designing prospective CMR myocarditis studies and the development of a more standardized imaging protocol in children.

## Abbreviations

CMR: Cardiovascular magnetic resonance
EGE: Early gadolinium enhancement
FPP: First-pass contrast perfusion
LGE: Late gadolinium enhancement
LV: Left ventricle
RV: Right ventricle
T2W: T2-weighted imaging
